# Exploring, Mapping, and Data Management Integration of Habitable Environments in Astrobiology

**DOI:** 10.3389/fmicb.2019.00147

**Published:** 2019-03-05

**Authors:** Marjorie A. Chan, Brenda B. Bowen, Frank A. Corsetti, William H. Farrand, Emily S. Law, Horton E. Newsom, Scott M. Perl, John R. Spear, David R. Thompson

**Affiliations:** ^1^Department of Geology and Geophysics, The University of Utah, Salt Lake City, UT, United States; ^2^Department of Earth Sciences, University of Southern California, Los Angeles, CA, United States; ^3^Space Science Institute, Boulder, CO, United States; ^4^Jet Propulsion Laboratory, California Institute of Technology, Pasadena, CA, United States; ^5^Department Earth and Planetary Sciences, University of New Mexico, Albuquerque, NM, United States; ^6^Department of Civil and Environmental Engineering, Colorado School of Mines, Golden, CO, United States

**Keywords:** astrobiology, habitable environments, authigenic minerals, Mars, cybertechnology, data management

## Abstract

New approaches to blending geoscience, planetary science, microbiology-geobiology/ecology, geoinformatics and cyberinfrastructure technology disciplines in a holistic effort can be transformative to astrobiology explorations. Over the last two decades, overwhelming orbital evidence has confirmed the abundance of authigenic (*in situ*, formed in place) minerals on Mars. On Earth, environments where authigenic minerals form provide a substrate for the preservation of microbial life. Similarly, extraterrestrial life is likely to be preserved where crustal minerals can record and preserve the biochemical mechanisms (i.e., biosignatures). The search for astrobiological evidence on Mars has focused on identifying past or present habitable environments – places that could support some semblance of life. Thus, authigenic minerals represent a promising habitable environment where extraterrestrial life could be recorded and potentially preserved over geologic time scales. Astrobiology research necessarily takes place over vastly different scales; from molecules to viruses and microbes to those of satellites and solar system exploration, but the differing scales of analyses are rarely connected quantitatively. The mismatch between the scales of these observations— from the macro- satellite mineralogical observations to the micro- microbial observations— limits the applicability of our astrobiological understanding as we search for records of life beyond Earth. Each-scale observation requires knowledge of the geologic context and the environmental parameters important for assessing habitability. Exploration efforts to search for extraterrestrial life should attempt to quantify both the geospatial context and the temporal/spatial relationships between microbial abundance and diversity within authigenic minerals at multiple scales, while assimilating resolutions from satellite observations to field measurements to microscopic analyses. Statistical measures, computer vision, and the geospatial synergy of Geographic Information Systems (GIS), can allow analyses of objective data-driven methods to locate, map, and predict where the “sweet spots” of habitable environments occur at multiple scales. This approach of science information architecture or an “Astrobiology Information System” can provide the necessary maps to guide researchers to discoveries via testing, visualizing, documenting, and collaborating on significant data relationships that will advance explorations for evidence of life in our solar system and beyond.

## Introduction

**FIGURE 1:**
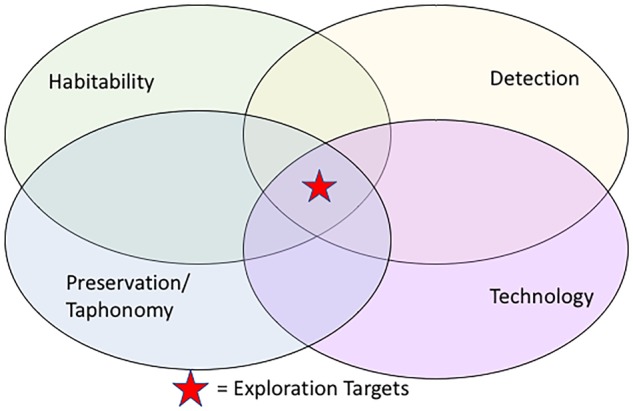
Scientific efforts to explore for biosignatures must link themes of habitability, preservation, and detection of biosignatures. The best potential for exploration targets lies at the intersection of science studies, and data-enabled cyberinfrastructure technology that can provide multi-scale data management and visualization.

In the interdisciplinary fields of geobiology, astrobiology, and NASA missions, one of the most important questions yet to be addressed is: *What are the best exploration strategies for finding extraterrestrial life?* Biosignatures (e.g., elemental, mineral, textural, or other scientific evidence of life) have mappable distributions at microscopic (<mm) to macroscopic (>km) levels. These scales of investigation can be linked via *probabilistic models*, statistical representations that represent dependencies permitting inference and prediction between the scales (Thompson et al., 2018b; Chan et al., 2018, 2019). Based on observations of Earth analogs, it is expected that extraterrestrial life also requires habitable environments – places where environmental conditions are conducive to sustain life (Preston and Dartnell, 2014; Cockell et al., 2017). Biosignatures are the records and/or remnants of life that must be preserved (study of taphonomy) in order to be detected (e.g., Westall et al., 2015). Detection of biosignatures requires tools and technology. The intersection of all these conditions (biosignature, preservation, detection, and technology) leads to the optimum chances for finding extraterrestrial life (Figure 1). In particular, harnessing the power of information technology (e.g., statistical measures and computer vision in an IT platform) grouped with scientific studies across multiple scales has great potential. For example, geostatistical modeling of relationships of habitable environments across scales and locations (e.g., Thompson et al., 2011), would help identify quantitatively-determined “sweet spots” to explore for extraterrestrial life (Candela et al., 2017) versus the primarily ad hoc methods in use today.

The relative “sweet spot” for habitability in this context relies on the preservation of biosignatures within minerals that have been in place since their original formation, as minerals are typically more robust to change over geologic time versus organic molecules. Rocks that were formed or had contact with fluids via ancient aqueous processes can contain biological information that future surface rovers can access. The notion of a habitable zone where records of former biotic activity once existed relies on the probability of defined biological and chemical variables. Quantification of the relationships among environmental variables (e.g., pH, salinity, permeability, etc.) that promote microbial life existence and preservation of biosignatures over geologic time on Earth, can then be used to quantify the probability of finding evidence of life at analogous sites on Mars.

Thus, this perspective paper proposes three focus strategies to search for extraterrestrial life on Mars where there are well established NASA missions with imagery and analysis data, and lithologic systems similar to Earth: (1) authigenic minerals (in place or *in situ*) as surface to near surface minerals; (2) context of habitable environments across multiple scales with quantification of geospatial relationships; and (3) use of cybertechnology and computer vision to help map and locate the most promising habitable environments for exploration. We further address what kinds of tools can be employed to address research in these collective areas.

Over the last few decades, overwhelming orbital evidence has confirmed the abundance of authigenic minerals on Mars. Authigenic minerals present a key location for preserving evidence of past life on other planets (Douglas, 2005). On Earth, environments where authigenic minerals form in the presence of water, these minerals are commonly associated with evidence of microbial life (e.g., Allen et al., 2004; Douglas, 2005; Fernández-Remolar and Knoll, 2008; Lowenstein et al., 2011; Preston et al., 2011; Allwood et al., 2013; Jahnke et al., 2014; Parenteau et al., 2014; Williams et al., 2015). Examples include cementing minerals forming where kinetics are facilitated by the metabolism or activities of microbes at sediment-fluid interfaces, or places where precipitation of minerals is associated with gradients in fluid chemistry where the right conditions exist to preserve the microbes that live in the substrate.

A challenge for astrobiological explorations is that the mismatch between the scales of these observations— from the macroscale satellite mineralogical observations to the microscale microbial observations— limits the applicability of our astrobiological understanding as we search for records of life beyond Earth. Here we present a concept of how future research might better bridge and link scales of observation by combining interdisciplinary geobiological data-driven analyses with new technologies: computer vision, including algorithms which automate and standardize object detection and quantify scene texture, and information technology more generally, which can combine and visualize disparate datasets in a statistically rigorous manner. The potential outcomes can generate valuable exploration maps to help narrow the search for extraterrestrial life, particularly relevant to Mars environments and mineralogy. Key advantages to integrated data management of astrobiology communication are: (1) the ability to increase, facilitate, and integrate communication and datasets of multiple subdisciplines; (2) limiting replication of data collection or unfruitful research paths because of shared, centralized data repositories; and (3) verification of important ideas and trends through data mining and data discovery.

## Methods Approach

### Search for Extraterrestrial Life

The last decade of planetary exploration has marked a new era of unprecedented discoveries that provide multiple lines of evidence for a rich history of water and preservation of sedimentary environments on Mars, and thus the potential for past or present life (e.g., Grotzinger and Milliken, 2012; Grotzinger et al., 2014; McLennan et al., 2014; Freissinet et al., 2015). Concurrently, there is growing evidence for water on other Earth-like planets orbiting other stars [e.g., from the Hubble Telescope and Kepler Missions (Kasting and Harman, 2013)]. Icy worlds with putative sub-surface liquid layers: Europa, Enceladus, and potentially Ceres also present new targets for astrobiologic investigation. A subsurface ocean has long been hypothesized for the Jovian satellite Europa and recent observations of possible geysers from Europa’s south pole (Roth et al., 2014) are strong evidence of possible subsurface liquid water. Cassini observed geysers emanating from the Saturnian moon Enceladus (Porco et al., 2006). Ceres observations from Dawn and Dawn combined with modeling indicate a possible past sub-surface ocean (Ermakov et al., 2017).

Among the most irrefutable proxies for extraterrestrial water are the authigenic minerals that waters leave behind in shallow subsurface critical zones (Brantley et al., 2007; Grant and Dietrich, 2017), where interactions between the lithosphere, hydrosphere, atmosphere, and potential biosphere all converge. It is crucial to understand how the presence of life can be preserved and detected as a biosignature in authigenic minerals in Earth environments so that the astrobiology community can develop detection criteria, and apply this knowledge to our explorations elsewhere in the universe.

### Potential Habitats on Mars

Widespread findings of fluid activity on Mars have been recorded through mineralogical, sedimentological, and geochemical evidence (McLennan et al., 2005; Bibring et al., 2006; McLennan and Grotzinger, 2008; Andrews-Hanna et al., 2010), which indicate potentially habitable environments on the planet. The assessment of past and present habitability on both a local and regional scale requires quantifying geologic features analogous to the resolution being investigated. Remote sensing instruments on orbiters, landers, and rovers sent to Mars have given scientists in-depth access to visual, mineralogical, and geochemical data at varying perspectives— allowing for a relatively complete geologic picture of the planet. Within the Martian rock record, evidence of biosignatures— if present— may have a low preservation potential due to factors such as surface ultraviolet and gamma radiation, oxidation, and extreme temperature fluctuations (among other issues). However, areas not affected by these issues, due to recent exhumation for example (Farley et al., 2013), can be identified.

It has been proposed that Mars in its beginning stages was a warmer and wetter planet (e.g., Farmer and Des Marais, 1999), when habitable regions may have been more widespread. Much work with the “follow the water” motto has suggested that if life existed on the surface of Mars in any fashion, it would have occurred in the Noachian and Hesperian periods where layered clays and sulfates originally formed (Tosca and Knoll, 2009; Hurowitz et al., 2010; Michalski et al., 2013). Should microbes exist or have existed on Mars, given conditions of surface radiation, changes in global temperatures, and stripping of surface layers by eolian processes? It is likely that microbial life might reside in the subsurface, at a depth and location stable for protection from harsh surface conditions (Perl et al., 2013). On Earth, the restrictions on the survivability of microbial life give us insight into the possible extent of planetary microbial life (Rothschild, 1990). A wide range of microbial-scale terrestrial life thrives in non-ideal environments and extreme conditions (Nealson, 1997; Allen et al., 2004; Douglas, 2005; Fernández-Remolar and Knoll, 2008; Lowenstein et al., 2011; Allwood et al., 2013; Jahnke et al., 2014).

The ability for microbes to exist and thrive, within an environment, as well as the existence of evidence of their activity in the form of organic matter or other biosignatures, suggests a favorable range of variables (i.e., a habitable environment) in place during the course of specific microorganism lifetimes. For example, the presence of authigenic minerals (e.g., sulfates, clays, etc.) over wide areas (Ehlmann et al., 2009; Michalski et al., 2013; Arvidson et al., 2014) observed via satellite (e.g., CRISM) on a macroscale provide evidence of widespread ancient aqueous alteration and diagenesis. This broad scale of observation can identify locations with water-related history that may have created favorable zones for biosignature preservation as well as regions that have experienced extensive diagenesis that may overprint these signatures. Indications of biosignature preservation at the mesoscale can be sought using tools on landers or rovers such as the MER Pancam and MSL Mastcam at an outcrop perspective (Berelson et al., 2011; Mata et al., 2012; Pepe-Ranney et al., 2012). Finally, when a rover encounters such authigenic minerals within its path, detection of Martian biosignatures on a microscale [e.g., MER (Mars Exploration Rover) Microscopic Imager (MI), MSL Mars Hand Lens Imager (MAHLI)] is possible. In this nested fashion, cues at the higher levels guide the investigation to the “needle in a haystack” of probable biosignatures.

## Results of Authigenic Minerals

Broadly, the search for life elsewhere can be subdivided into two main foci: the search for extant life, and the search for past life. Both approaches require knowledge of where life can exist, whereas the search for past life also requires that an element of preservation (i.e., fossilization potential) be inherent to the environment where life once existed. As described below, authigenic minerals form in place and have the potential to preserve biosignatures over geologic time scales. Astrobiological science must focus on the systems that have the strongest potential to preserve biosignatures across geologic time scales, and consider the relationships and environmental parameters that control their precise location. On Earth, microbial life exists in fluid-filled pore spaces from the surface to the deep subsurface.

**FIGURE 2:**
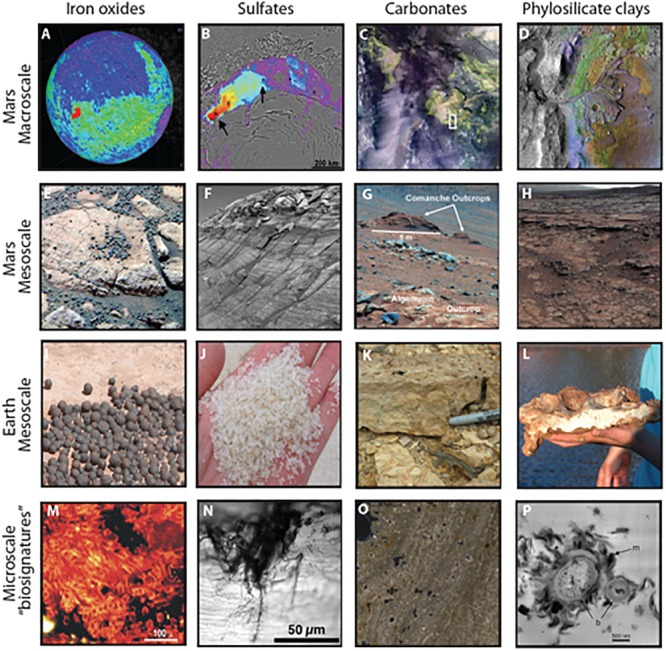
Examples of morphological biosignatures within authigenic mineral systems identified on Mars. **(A)** Hematite (red) in Meridiani Planum region detected by TES (after Christensen et al., 2000). **(B)** Sulfate (red) in the polar dunes detected by OMEGA (NASA/JPL-Caltech/JHUAPL/Brown University). **(C)** Carbonate-bearing units (green) in Nili Fossae region detected by CRISM (Ehlmann et al., 2008). **(D)** Phyllosilicate clays (bright colors) associated with delta deposit at Jezero crater detected by CRISM (NASA/JPL/JHUAPL/MSSS/Brown University). **(E)** Hematite concretions at Eagle Crater, Opportunity (NASA/JPL/USGS). **(F)** Sulfate-cemented sandstone at Endurance Crater, Opportunity (NASA/JPL/Cornell). **(G)** Carbonate-containing outcrops, Spirit (NASA/JPL-Caltch/Cornell) (Morris et al., 2010). **(H)** Clay-rich mudstone and sandstone at Yellowknife Bay, Curiosity (NASA/JPL-Caltech/MSSS). **(I)** Hematite concretions from the Jurassic Navajo Sandstone (Chan et al., 2004, 2005). **(J)** Gypsum sand grains from dunes surrounding acid saline lakes in Western Australia. **(K)** Carbonate stromatolite from the Eocene Green River Formation. **(L)** Clay bed (kaolinite and halloysite) from acid saline lake sediments. **(M)** Algal/fungal filaments within 2.1 million year old iron oxide precipitates at Rio Tinto in Spain (Fernández-Remolar and Knoll, 2008). **(N)** Microbial remnants within gypsum crystal in Western Australia (Benison et al., 2008). **(O)** Stromatolite laminae from the Green River Formation with quartz grains trapped at high angle of dip indicating the former presence of sticky microbial mat. **(P)** Bacteria lined with clay minerals from tephra in Hawaii (Leveille and Konhauser, 2007).

Authigenic minerals are those that form in place (*in situ*) and have the potential to record water and environmental conditions of the critical zone where life flourishes, in contrast to minerals transported into a site. Authigenic minerals are common in terrestrial sedimentary systems and can be used to constrain geochemical and environmental parameters of their formation (e.g., pH, temperature, pressure, aridity, etc.). We focus on four important authigenic mineral systems that are preserved on Mars and indicate the potential for watery environments: iron and manganese oxides, sulfates, clays, and carbonates (Figure 2 and Table 1). On Earth, biosignatures have been identified in each of these authigenic mineral systems, with the most well studied occurring in marine carbonates (e.g., Grotzinger and Knoll, 1999).

**Table 1:** Authigenic minerals for study of biosignature preservation potential and environmental relationships.

Scales	Length range	Example observations	Example Mars observations
Macro	Kilometers to meters	Remote sensing, aerial photography, imaging	Orbiters
Meso	Meters to millimeters	Field (modern and ancient), outcrop facies	Rovers, HiRISE images from Mars Reconnaissance Orbiter
Micro	Millimeter to nano	Microscopy, mineralogy, microbial ecology	Rover analyses

In studying Mars analog authigenic minerals on Earth, researchers employ multiple approaches to characterizing microbial communities. Various approaches examine presence/absence, type and genetic potential for biosignature production, ecological occurrences, and interactions with authigenic minerals (e.g., mineralogy compositions, chemistry/trace elements, textures) at multiple sites with active authigenic mineral formation. These types of studies will enable the understanding of geobiological relationships within authigenic mineral groups. This includes environments where these minerals are actively forming in modern to geologically young settings, in order to provide spatial variations in initial and early diagenetic biosignature preservation processes.

### Iron and Manganese Oxides

Iron oxide minerals are common in Earth systems (Christensen et al., 2000; Schwertmann and Cornell, 2008) and widespread on Mars (e.g., Table 1) as both nanophase dust and authigenic precipitates. The geologic record of terrestrial iron oxides extends from the Archean, 3.8 billion years ago (e.g., Precambrian Banded Iron Formation) to modern deposits of rust wherever iron is exposed to air and moisture. These minerals reflect specific redox and pH chemistry, and are commonly tied to microbial metabolism, including reductive and oxidative processes (Ortoleva, 1984, 1994; Cornell and Schwertmann, 1996; Konhauser et al., 2011). The potential for long-term biosignature preservation potential of iron oxides has been questioned (Sumner, 2004), however, unambiguous preservation of cells in iron and manganese oxide deposits has been documented from hot springs (Wade et al., 1999; Parenteau and Cady, 2010), acid mine drainage precipitates (Fernández-Remolar et al., 2005; Fernández-Remolar and Knoll, 2008; Williams et al., 2017) and rock coatings (e.g., Allen et al., 2004). These studies demonstrate that microbial cells can be ‘entombed’ or ‘permineralized’ by iron or manganese oxides and preserved along with the chemical environmental information within the minerals themselves. Manganese oxides have also been reported on Mars (Arvidson et al., 2016; Lanza et al., 2016). Manganese oxides are mobilized under oxidizing conditions similar to Precambrian Earth and are important indicators of a past environment that might have been favorable to life.

### Sulfates

Sulfates are abundant in aqueous low temperature (e.g., evaporite and weathering) settings on Earth (Alpers et al., 2000). Several types of sulfate minerals including Mg/Fe mono- and poly-hydrated sulfates have been identified on Mars as eolian surface accumulations (Langevin et al., 2005), as a widespread component of lithified sedimentary outcrops with remnant lacustrine and dune/interdune deposits (Klingelhöfer et al., 2004; Bibring et al., 2006; Milliken et al., 2008; Farrand et al., 2009), and as fracture-filling veins of calcium sulfate at both Endeavor and Gale craters (Squyres et al., 2012; Nachon et al., 2014). Varying mineralogical, chemical and textural characteristics of sulfates relate to environmental parameters such as temperature, pH, oxidation, weathering conditions, and host brine composition, and can provide a wealth of information about past environmental conditions. The details of how biosignatures and organic compounds are preserved in sulfates are poorly understood (Aubrey et al., 2006). Although there is limited evidence of microbial metabolism involved in facilitating direct precipitation of sulfate minerals, microbes are still involved in some steps of sulfur cycling (e.g., responsible for oxidizing sulfur to form sulfate minerals) (Benison and Bowen, 2013). While once thought to be a poor archive of microfossil material, recent discoveries of fossilized cells in gypsum deposits from the Alps of Northern Italy illustrate the potential for preservation (Schopf et al., 2010). The presence of acid sulfates on Mars has been inferred to suggest that past aqueous environments may have been inhospitable. However, extremely acid (pH < 4) hypersaline environments have been shown to host diverse microbial communities (Mormile et al., 2009) and biosignatures are preserved within evaporite (halite and sulfate) minerals forming in those environments (Benison et al., 2008; Lowenstein et al., 2011). Modern gypsum crusts from the hyper-arid Atacama Desert in Chile also preserve microbial cells and highlight the ability of halophiles and endoliths to survive under conditions of very low water activity (Wierzchos et al., 2011). In addition, recent studies demonstrate the potential for biosignature preservation in gypsum from ancient stromatolites (Allwood et al., 2013) to modern gypsum crusts (Sahl et al., 2008a; Jahnke et al., 2014). Detection of lipid biomarkers in hyperarid soils in the Atacama Desert (Wilhelm et al., 2017) also demonstrate the potential for preservation of organics in sulfates.

### Carbonates

Carbonates have long been studied for their preservation of biosignatures as the conditions and processes that foster carbonate precipitation are intrinsically linked to the carbon cycle. On Earth, carbonates form in a range of aqueous environments including lacustrine, shallow marine, springs, and within subsurface diagenetic settings. In paleoenvironmental communities, carbonates are considered excellent records of past conditions. Carbonates have long been considered a “holy grail” of astrobiology on Mars, as their occurrence as precipitates would suggest a CO_2_-rich atmosphere and neutral to moderately alkaline fluid chemistry. However, carbonates generated from diagenesis of basalts might not yield as robust of an environmental signal for biosignature preservation as chemical or biochemical precipitated carbonates. While apparently not widespread on the surface, carbonates have now been identified in several locations on Mars (Ehlmann et al., 2008; Niles et al., 2013; Wray et al., 2016). Some documented carbonate discoveries include the Nili Fosse region (Ehlmann et al., 2008), the Columbia Hills of Gusev crater (e.g., Morris et al., 2010), Columbus crater (Wray et al., 2011), Leighton crater (Michalski and Niles, 2010), McLaughlin Crater (Michalski et al., 2013), and Huygens crater (Wray et al., 2016). New discoveries in Gale crater highlight the importance of finding preserved organics on Mars that could be relevant to carbonates as well as other mineralogies (Freissinet et al., 2015; Eigenbrode et al., 2018).

### Clays

Clays, in this discussion, are a group of phyllosilicates (vs. the grain size usage of <0.002 mm) where the mineral structure is composed of sheets of silica-oxygen tetrahedra with an inter-sheet capacity to incorporate other ions, water, and organics. Clays are significant indicators of surface processes forming in the presence of compressed water vapor at a temperature of several hundred degrees centigrade, to ambient water temperatures common at the Earth’s surface (Kerr, 1955). Typically, clays are alteration products resulting from rock weathering and are, compositionally, a major mineral component of the Earth’s soil environment (Wilson, 1999). Some clay petrogenesis results from the passive nucleation of amorphous silicates on bacterial cells that later transforms to more crystalline phases (Konhauser and Urrutia, 1999). Due to their high potential for concentration and preservation of organic compounds, deposits rich in clay minerals and other fine-grained sediments, are prime targets in the search for organic remnants of life on Mars (Konhauser et al., 2002; Ehlmann et al., 2008). The presence of clays is now well documented on the surface of Mars and represents water-rich environments that have been proposed as some of the most promising locations for biosignature preservation (Bibring et al., 2005; Poulet et al., 2005; Ehlmann et al., 2011).

## Discussion

### Scales and Context

Scales and context of biosignatures on Earth have analogous approaches in explorations such as on Mars. Terrestrial studies of biosignature preservation in multiple authigenic mineral systems should use a systematic approach and consistent instrument toolbox from macro- (>km), to meso- (m), to microscale (<mm) (Table 2) to help identify areas with the range of both good and poor biosignature preservation. Examinations at multiple scales is important because mission explorations need flexibility to go between coarse remote scales down to rover sub-millimeter scales.

**Table 2:** Scales of study for biosignatures and example observational approaches.

Mineral system	Example authigenic minerals	Example Mars sites	Mars example reference
Oxides’	Hydrous ferric oxides: HFO	Meridiani Planum	Christensen et al., 2001;
	Goethite: FeOOH	Aram Chaos	Klingelhöfer et al., 2004;
	Hematite: Fe_2_o_3_	Gale Crater	Bibring et al., 2006;
	Manganese oxides: MnO		
	Pyrolusite: Mno_2_		Farrand et al., 2009
Sulfates	Gypsum: CaSo_4_^.^2H_2_0	Polar region	Gendrin et al., 2005;
	Bassanite:2CaSo_4_(H_2_0)	Meridiani Planum	Langevin et al., 2005;
	Jarosite: NaFe_3_(S0_4_)_2_(OH)_6_	Gale Crater	Milliken et al., 2008;
	Alunite: KAI_3_(S0_4_)_2_(OH)_6_	Mawrth Vallis	Swayze et al., 2008;
			Farrand et al., 2009;
			Ehlmann, 2010;
			Wray et al., 2010
Carbonates	Calcite: CaCO_3_	Nili Fossae	Ehlmann et al., 2008;
	Magnesite: MgCO_3_	Columbia Hills	Michalski and Niles, 2010;
	Siderite: FeCO_3_	Huygens Crater	Morris et al., 2010;
	Ankerite: Ca(Fe,Mg,Mn)(CO_3_)_2_	Noachis Terra	Wray et al., 2011;
			Michalski et al., 2013
Clays	Nontronite, Saponite	Jezero Crater	Poulet et al., 2005;
	Montmorillonite: (Na, Ca)_0.33_	Columbia Hills	Bishop et al., 2008;
	(Al, Mg)_2_(Si_4_O_10_) (OH)_2_	Gale Crater	Mustard et al., 2008;
	Kaolinite/halloysite	Endeavour Crater	Ehlmann et al., 2009;
	Al_2_Si_2_O_5_(OH)_4_	Mawrth Vallis	Wray et al., 2009
	illite KAl_2_ AlSi_3_O_10_(OH)_2_		

*This is not an exhaustive study of each mineral system, but shows a consistent approach to relevant minerals with similar counterparts on Mars. Mars examples modified after Ehlmann (2010)*.

Studies at the macroscale provide broad perspectives of terrain and mineralogy that are comparable to satellite imagery observations. This context frames mesoscale “rover-scale” field studies including terrestrial analog field data (georeferenced landscape aerial gigapan images, environmental maps of facies, surface geochemistry, etc.), digital elevation models, and field-based spectroscopic mineral mapping. New technology for exploring the huge imaging volumes produced by ongoing rover missions include virtual reality headsets, such as the Microsoft Hololens with the Jet Propulsion Laboratory (JPL), OnSight software (Adair et al., 2018; Stein et al., 2018). Mesoscale studies provide the context for the detailed observations of microscopy, mineralogy, and ecology within microscale studies of authigenic minerals (Tables 2, 3).

**Table 3:** Macro to microscale data in biosignature studies require field, lab, and analogous planetary mission instrumentation (satellite/rover) to evaluate measures of habitability.

SCALE	Scientific data objectives	Field instruments for earth evaluation	Lab instruments for earth evaluation	Planetary instruments for evaluation
**MACRO**	Context Terrain	–	Maps, Google Earth	MRO
	Hi Res. Satellite Images	UAVs	DEM	MRO HiRISE
	Mineralogy	Field Spectrometer	AVIRIS-C AVIRIS-NG, ASTER, WorldView-2, 3	MRO CRISM
	Location, altitude	Laser Range Finder, GPS	Georeferencing	MOLA

**MESO**	Microbial ecology	Frozen DNA Habitat sunlight, season, exposure, aspect, etc.	PCR amplification, MiSeq and or HiSeq	Needs future development
	Microbe sampling	Commercial kits and protocols (e.g., Zymo Xpedition; DNA FastPrep; RNeasy isolation)	Synthesize cDNA from RNA using random hexamer primers and SuperScript III first-strand synthesis kits	Needs future development
	Petrology	Hand lens, Sampling	Petrography (authigenic vs. host)	MI (MER), MAHLI (MSL), MECA-AFM (Phoenix)
	Mineralogy, pore geometries	Field Spectrometer	XRD, QEMScan	CheMin, Pancam, MastCam, Mini-TES
	Chemistry	Handheld LIBS	–	APXS, ChemCam, SAM
	Petrophysical	*In situ* Minipermeameter	Plug measurements (porosity, permeability)	MECA-wet chemistry (Phoenix)
	Spatial relationships	Stratigraphic sections, depth spatial sampling	–	Mastcam images for stratigraphic sections
	Field Context	Field Imagery, Gigapans, Laser Range finder	–	Mastcam, Pancam
	Field Context	Facies, geologic map	–	Facies, Geologic maps

**MICRO**	Elements	Handheld XRF/LIBS	Whole Rock LA-ICP-MS	ChemCam
	Isotopic Chemistry	–	SIMS, SIRMS	Viking GCMS to SAM (MSL)
	Volatiles	–	micro-reflectance infrared	SAM (MSL)
	H, H/D, Li, B C, O, S detection, trace elements	LIBS	IC, stable isotopes, SEM/EMPA, SIRMS	ChemCam, SAM
	DNA and RNA	–	PCR amplification; MiSeq, HiSeq, and/or MinION DNA sequencing	–
	Microscopic Textures	Field Raman, XRD, petrography	QEMScan, SEM/EMPA, TEM, Raman, fluid experiments	MAHLI, MI, MECA-AFM (Phoenix)

**ALL**	Spatial statistics, Geospatial Visualization	Interdisciplinary, Cross cutting	Geostatistics, Computer Vision, segmentation, Data Management GIS-based platform	Needs future development, to synthesize across all scales

*All abbreviations are defined the text and also in Table 4*.

#### Macroscale Context: Remote Sensing Reconnaissance and Mineral Mapping

Remote sensing data represent macroscale information on potentially habitable environments. These datasets give a regional context for habitability studies and can be used to target the most promising areas within the terrestrial analog sites for more detailed mesoscale to microscale studies. The characterization of terrains using remote sensing data has been improved through new remote sensing technologies such as hyperspectral high spatial resolution imaging, LIDAR (light detection and ranging), and combined high spatial resolution imagery with expanded multispectral coverage. Hyperspectral remote sensing datasets, are similar to the data collected by the OMEGA and CRISM imaging spectrometers aboard the Mars Express and Mars Reconnaissance Orbiter spacecraft respectively. Mars sensors have observed vibrational overtone absorption features associated with phyllosilicate minerals and isolated occurrences of carbonate overtone features in Noachian-aged terrains (Bibring et al., 2005; Mustard et al., 2008; Niles et al., 2013) and water and sulfate overtone features in early Hesperian terrains (Bibring et al., 2005; Gendrin et al., 2005). Terrestrial airborne imaging spectroscopy using platforms such as NASA’s “Classic” and “Next Generation” Airborne Visible Infrared Imaging Spectrometers, AVIRIS-C (Green et al., 1998) and -NG (Thompson et al., 2018a) respectively, have been used to map sites of hydrothermal alteration and unique sedimentary environments (e.g., Kruse, 1988; Crowley, 1993; Thompson et al., 2018b). Such datasets have also been used to detect minerals generated through microbiologic activity (e.g., Anderson and Robbins, 1998).

High spatial resolution panchromatic imagery (sensors such as GeoEye and WorldView-1) and high spatial resolution extended multispectral imagery (i.e., multispectral datasets with more than 4 bands; e.g., WorldView-2 and 3) have had a profound impact on terrestrial remote sensing studies both through the ability to spatially resolve geomorphologic features that were previously unresolvable, as well as to extrapolate some spectroscopic analyses to extended multispectral datasets and thus improve the detectability of materials at smaller spatial scales (Farrand et al., 2013). These datasets can be considered analogous to the high spatial resolution three-color imagery collected by the MRO HiRISE sensor, and provide a context template for mapping preservation potential at regional scales.

#### Mesoscale Systems: Geologic Outcrops

Key mineral systems (e.g., oxides, sulfates, carbonates, and clays) investigated within their geologic context in environments where they are actively precipitating provide insights into the range of habitable conditions where evidence of life may be preserved. Many tools (Tables 3, 4) to derive measures of habitability and geologic preservation of biosignatures in authigenic minerals are critical for characterizing geologic environments in the context of authigenic mineral formation processes and microbial ecology.

**Table 4:** Instrumentation Abbreviations.

AIS	Astrobiology Information System
APXS	Alpha-Particle X-ray Spectrometer
ASTER	Advanced Spaceborne Thermal Emission and Reflection Radiometer
AVIRIS	Airborne Visible/Infrared Imaging Spectrometer (-C = “Classic”, -NG = “Next Generation”)
ChemCam	Chemical Camera
CheMin	Chemistry and Mineralogy
CRISM	Compact Reconnaissance Imaging Spectrometer for Mars
DEM	Digital Elevation Models
EMPA	Electron Microprobe Analysis
ENVI	ENvironment for Visualizing Images
GIS	Geographic Information Systems
GPS	Global Positioning System
HiRISE	High Resolution Imaging Science Experiment
HSI	Hyperspectral Imagery
IC	Ion Chromatography
ICPMS	Inductively Coupled Plasma Mass Spectrometry
IR	Infrared
JPL	Jet Propulsion Laboratory
LIBS	Laser-Induced Breakdown Spectroscopy
LIDAR	Light Detection and Ranging
LMMP	Lunar Mapping and Modeling Portal
MAHLI	Mars Hand Lens Imager
MastCam	The Mars Science Laboratory (MSL) Mast Camera
MER	Mars Opportunity Rover
MG-RAST	Metagenomic Rapid Annotations using Subsystems Technology
MI	Microscopic Imager
MOLA	Mars Orbiter Laser Altimeter
MRO	Mars Reconnaissance Orbiter
MSL	Mars Science Laboratory
NAI	NASA Astrobiology Institute
NIR	Near Infrared
Pancam	Panoramic Camera
PCR	Polymerase Chain Reaction
QEMSCAN	Quantitative Evaluation of Minerals by SCANning electron microscopy
RAT	Rock Abrasion Tool
REE	Rare Earth Element
SAM	Sample Analysis at Mars
SEM-EDS	Scanning electron microscopy – Electron Diffraction System
SIMS	Secondary Ion Mass Spectrometry
SIRMS	Stable Isotope Ratio Mass Spectrometer
TEM	Transmission Electron Microscopy
TES	Thermal Emission Spectrometer
UAV	Unmanned Aerial Vehicles
VNIR	Visible and Near-Infrared
XRD	X-ray diffraction
XRF	X-ray fluorescence
μ-R-IR	Micro-Reflectance Infrared

The environmental context for the targeted authigenic minerals (Table 3) requires basic field studies such as: (1) defining, mapping and sampling of depositional facies based on field observations and interpretations from gigapans (high-resolution panoramic photographs); (2) creating measured stratigraphic sections that include characterization of stratigraphic architecture and spectral stratigraphy through field reflectance measurements; (3) characterizing spatial patterns in both detrital and authigenic mineralogy and geochemistry; and (4) sampling and characterizing the geochemical fluids associated with authigenic minerals (e.g., salinity, temperature, pH, and DNA/RNA extraction). Petrophysical properties (e.g., porosity and permeability) are also important measures. Field-based observations of fluid chemistry and visual heterogeneities will help to identify areas where environmental (pH, temperature, salinity, etc.) gradients exist and where transitions between authigenic minerals likely occur.

After a surface exploration mission is underway, many critical tactical decisions about where to focus and collect samples are made at the scale of outcrops, often with limited prior information and the constant time pressure of an advancing mission (Thompson et al., 2015; Doran et al., 2016). Records of systematic sampling can help to inform these decisions.

#### Microscale Detection: Fluids, Minerals, and Microbes

Current research examining microscale detection of potential biosignatures has limitations related to the small scale of materials and instrumentation. Typical studies examine the authigenic minerals formed by diagenetic, sedimentary, aqueous and hydrothermal processes. Thus, attempts to study the fluids associated with the minerals, and testing for microbes involve water chemistry, petrology and geochemistry, and molecular microbial community analyses. Basic characterization of the aqueous chemistry of fluids associated with the authigenic minerals have implications for where life might have existed. Example approaches are shown in Table 3 (with abbreviation notations in Table 4). Alteration minerals are detectable through IR mapping (Greenberger et al., 2015) as well as via micro-reflectance infrared (μ-R-IR) spectroscopy (King et al., 2004) that is extremely sensitive to detecting even poorly crystalline clay minerals (Farrand and Singer, 1992; Vicenzi and Heaney, 2000) or possible carbonaceous minerals and hydrous iron oxide minerals (e.g., Bishop et al., 2013; Muttik et al., 2013, 2014) on the surface of Mars.

The stable isotope composition of authigenic minerals reflects the origin of fluid and its temperature (δ^7^Li, δD, δ^18^O, and δ^17^O), salinity (δ^11^B) and fluid recycling during rock-water interaction (δ^7^Li, Fe-content of the alteration material) and involvement of bacterial/thermochemical processes (δ^34^S, B). Lithium and boron stable isotopes are potential sensitive tracers of fluid-rock interactions in any system (Hervig et al., 2002; Williams and Hervig, 2002; Deyhle and Kopf, 2005; Gonfiantini and Pennisi, 2006; Williams et al., 2007; Vigier et al., 2008; Pennisi et al., 2009) especially in phyllosilicates. These isotopes can constrain the compositions of aqueous crustal fluids, as previously applied to impact crater materials (Williams et al., 2007; Muttik et al., 2011) and burial diagenetic bentonite (Williams et al., 2013). Hydrogen and oxygen isotope geochemistry of secondary clay minerals is widely used for interpretation of the origin and evolution of alteration fluids, mineral-water interaction and determination of temperatures at the time of formation of secondary minerals in a hydrothermal system (e.g., Muttik et al., 2010; Gilmour et al., 2013).

Fundamental to answering the question of where and how evidence of life is preserved in authigenic minerals, is to examine the microbial communities associated with them today, and what may have been associated with their formation in the past. This addresses the imperative question of what organisms have the potential to leave behind as biosignatures that can be associated with authigenic minerals (e.g., Spear et al., 2007; Sahl et al., 2008b; Lowenstein et al., 2011). Microbiota from all three domains of life coat rocks, live within the rocks, weather rocks, produce and degrade minerals, and are instrumental to the ‘rock cycle’ as well as the cycling of nearly every element (Spear and Corsetti, 2013). Pore space within a rock can be home to a complex microbial endolithic ecosystem with photosynthetic autotrophs providing fixed carbon to heterotrophs while at the same time altering the mineral structure (Walker et al., 2005; Walker and Pace, 2007).

**FIGURE 3:**
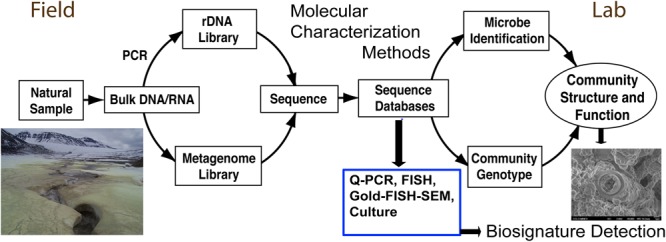
Microbial analysis is typically characterized in the field context with sample collection **(left)**, for further analyses and culturing in the laboratory leading to the eventual biosignature detection **(right)**. Currently, modern environmental samples are more conducive to biosignature detection than ancient samples that lack good preservation of DNA.

Molecular microbial community analysis with DNA/RNA sequencing technology in modern systems is well established (e.g., Illumina MiSeq and HiSeq DNA sequencing). Environmental parameters of sunlight, season, exposure and aspect, can all have an effect on the microbial communities associated with rocks and minerals (Walker et al., 2005; Walker and Pace, 2007). For terrestrial fieldwork, DNA and RNA is typically extracted from selected authigenic mineral samples and frozen for transport back to the laboratory (Figure 3). There, DNA can be subjected to PCR amplification of the 16S rRNA gene in a sample-specific, bar-coded approach [via the Illumina sequencing platform (MiSeq and or HiSeq)] to determine the microbial community composition, the ‘who is there,’ of each individual sample. More recently, work has been done to establish methods to characterize the active community via ribosomal RNA content (Jones and Lennon, 2010). However, what is lacking are large sample numbers and analyses in combination with measured geologic context. Furthermore, finding ancient preserved DNA in lithified rocks is much more challenging. Yet, these kinds of modern analyses are sorely needed because the community parameters can yield a strong data set for statistical evaluation in evaluating where the best habitable environments occur and where detailed mineralogy and petrology can be informed. Certainly more sophisticated and more comprehensive approaches will follow as instrumentation and technology rapidly changes.

**FIGURE 4:**
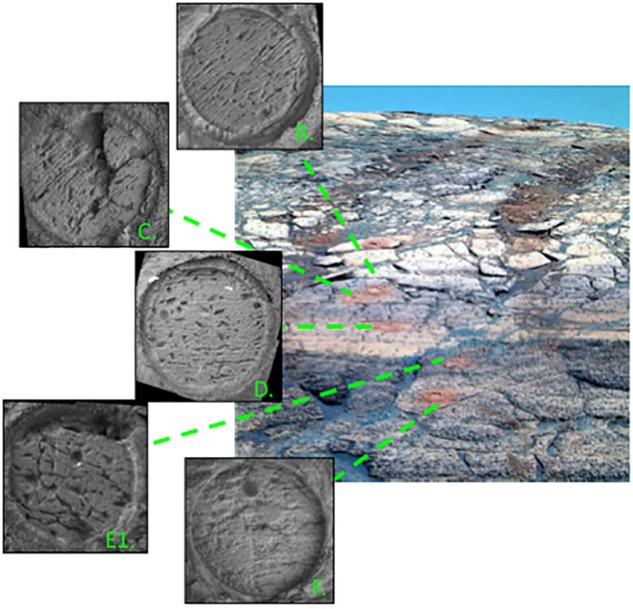
Microscopic Imager (MI) mosaics of abraded rocks in the Karatepe stratigraphic section in Endurance crater, Meridiani Planum, Mars. Three color Pancam composite images show overview of the stratigraphic section with locations of MI mosaics (inset images) indicated by dashed green lines (Herkenhoff et al., 2004). Endurance crater is the only site to host all types of secondary pores observed (Perl and McLennan, 2008). Letter notations match the Burns formation schematic of Grotzinger et al. (2005).

Other possible approaches to understanding microbial life in authigenic minerals includes building microscale laboratory fluid-rock-microbe experiments. For example, unique laboratory experiments can investigate the preservation potential of microbes that could remain within secondary porosity after periodic groundwater recharge events, as described by McLennan et al. (2005) and modeled after the “wetting upward” eolian to interdune system defined by Grotzinger et al. (2005). Upon abrasion of rocks within the outcrop outlining Endurance Crater (Figure 4), discovered void spaces were interpreted as secondary pore space created from the dissolution of soluble minerals from groundwater movement that could constrain water activity and could act as microscale reservoirs for organic material (Perl et al., 2013). Such pores could act as a semi-closed system with access to brines for organics to occupy while buried far enough away from the surface to avoid destructive interference from solar/UV radiation (Sumner, 2004). Summons et al. (2011) note that preservation is optimized with low temperatures and near-zero permeabilities.

### Harnessing Cybertechnology for an Astrobiology Information System (AIS)

**FIGURE 5:**
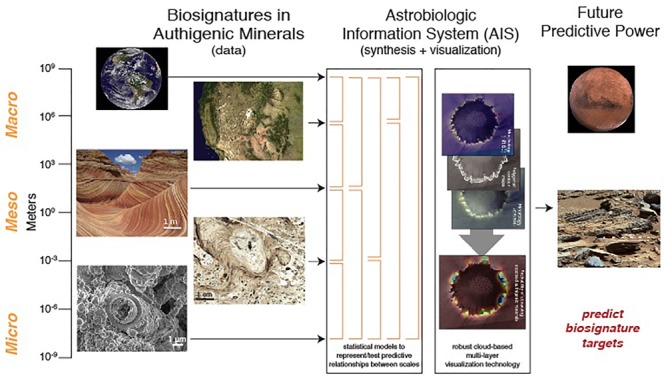
An integrative AIS looks toward predictive power for futures missions to map habitable environments and find the “sweet spots” of biosignatures through (1) the infusion of geologic and biologic characterizations at multiple scales with (2) statistical measures and a visualization platform.

Ultimately, all the multidimensional data for astrobiology studies could greatly benefit from a community-accepted data management system to allow important and statistically correlated relationships between all the different data types as different layers (individual, collective and/or superimposed). Community input is extremely important in developing a shared database that efficiently captures the workflow of the community it serves (e.g., Chan et al., 2016). Researchers that use multiple databases may also have invaluable suggestions on how to integrate communication and transfer between systems more seamlessly. Systematic characterization is needed across multiple mineral systems in order to provide a fundamental framework for quantifying measures and probability of habitability (Figure 5). Various mineral, habitability map layers, and environmental parameter visualizations would enable users to express and present data in new and advanced ways, and discern what patterns and parameters are capable of preserving signatures of microbial life for better predictive algorithms in both Earth and Mars examples. This is essentially a spatially layered geographic information system that could be called an Astrobiology Information System (AIS). Synthesizing all the data sets from macro to micro scales would allow for a new approach toward evaluating the processes at each of these scales and within the various target mineral systems that facilitate habitation and preservation of biosignatures.

**FIGURE 6:**
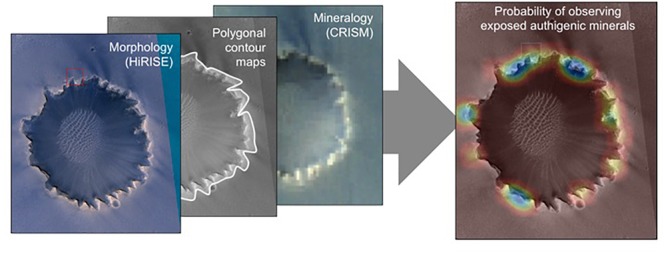
Conceptual illustration of integration of geo-registered datasets at Victoria Crater on Mars to produce map overlays indicating the probability of target variables driving exploration and sampling decisions. In the panel at right, cool colors indicate higher probability of discovering exposed authigenic minerals at that location. Crater diameter ∼800 m across. HIRISE Credit: NASA/JPL/University of Arizona. CRISM Credit: NASA/JPL/JHUAPL.

Two modern computer science technologies are critical to an AIS: automated instrument data analysis, and multi-scale spatiostatistical models that relate them. Automated instrument analysis includes machine vision, with tools that supplement traditional research and analysis techniques to identify morphological structures and positions. Virtual Reality analysis of 3D data sets is currently done by human interpretation, for example in the discovery of mudcracks in Gale crater (Stein et al., 2018). Future computer analysis techniques may include other sophisticated 3D analyses. Image-based relationships are automated consistently and from large catalogs, so that the attributes can participate with other quantitative measurements in the subsequent statistical analyses (Figure 6). Thus, new algorithms and artificial intelligence can find and recognize image patterns at multiple scales: from the micro-scale, where biogenic structures may be visible; to the mesoscale, where geologic fabrics and boundaries indicate compositional units with common properties; to the macro-scale, where wide-area physical processes indicate regions where preservation potential is greatest. Spectroscopic methods for automated mapping, characterization, and anomaly detection allow automatic pattern identification in similar fashion (Thompson et al., 2015, 2018b) to interpret mineralogy. A third important component is a geostatistical model capable of inferring spatial and contextual relationships from measurements made at multiple scales (Thompson et al., 2018b). This can also integrate: standard probabilistic models drawn from geostatistics (Cressie and Cassie, 1993); factor analysis, which decomposes a dataset into component trends that may vary independently, and may imply different physical effects (Bishop, 2006); and potentially latent semantic analysis, which infers the pure “topics” represented by a dataset, even if no single instance expresses just one (Blei et al., 2003). All of these in combination can produce statistical analyses and quantitative habitability predictions to visualize and map where habitable environment may be present. An integrated geographic information, data management, and visualization system will allow scientists to interact with data and results and will ingest, store, manage, and distribute large quantities of multi-dimensional science data and interdisciplinary results (data sets, algorithms, models, and workflows) produced by many interdisciplinary scientists.

The blend of geoscience, planetary science, microbiology/geobiology/ecology, geoinformatics and cyberinfrastructure technology disciplines in a holistic effort will be a major paradigm shift for astrobiology (Figure 5). The synthesis of biosignatures in authigenic mineral environments with probabilistic models will allow correlation of diverse datasets to establish primary and previously unrecognized relationships between biologic and geologic parameters on all scales. Geologic and biologic data can be digitally converted to map data that retains scientific fidelity in 3D (latitude, longitude, depth) as well as adding a 4th dimension of time. The science information architecture will enable researchers to discover, test, visualize, document, and collaborate on significant data relationships that will advance evidence of whether life might exist in other planetary environments. An AIS can affect sampling strategies, analytical techniques, detection techniques, and presentation of data.

The single, simple user platform (with some similarities to Google Earth) can encapsulate the entire interdisciplinary design as a portal to assimilate the data results or lessons learned by the collective research. This is the essential step that will help generate new knowledge. Like many other aspects of our digital age, this will allow new science discoveries and increase knowledge exchange, to create a geospatial and temporal model that will influence all aspects of NAI. An AIS system has the potential to reach many constituents and stakeholders, and can have far reaching effects on the future of NAI and astrobiology.

Existing GIS platforms with geospatial visualization capable of aggregating large volumes of planetary science data include JPL’s Solar System Treks Project (SSTP)^1^ (Law and Solar Systems Trek Team, 2018). SSTP is a family of web portals that provides visualization and analysis tools. LMMP, the former lunar portal, is now Moon Trek^2^, Mars Trek^3^, Vesta Trek^4^ and Ceres Trek^5^ are also publicly available. These highly successful GIS-based portals provide a geospatial framework and infrastructure developed for NASA missions and data. In these portals, maps and images with science data allow for easy viewing or toggling of geospatial and temporally referenced data from one layer to another to visually identify relationships in the data. Interactive machine learning based analysis tools are also available to support research. Expanding these concepts to the astrobiology community so individual researchers could upload and access new data would allow rich information sharing, and could provide tools for users to visualize and analyze data and content through innovative methods of synthesizing disparate datasets.

**FIGURE 7:**
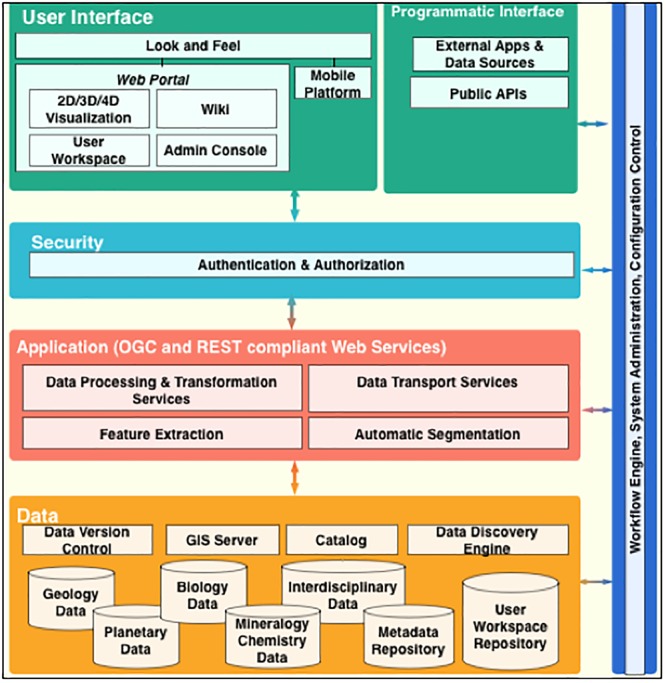
Conceptual AIS data system architecture that could integrate user workflow and astrobiologic data and spatial context across multiple scales.

A conceptual AIS data system for the astrobiology community is depicted in Figure 7 and will allow:

1. Collection and cataloging of data products, findings, and associated scholarly publications.
2. Processing data into image mosaics and map layers for scientific visualization (e.g., maps of sedimentary rocks, depositional environments, specific minerals, different elements, etc.).
3. Community viewing of the data in multiple scales, dimensions and correlations to compare observations and highlight trends.
4. Map functions like drawing contour labels to map the surfaces, and annotation by providing key classifications such as mineralogical and morphological categories for every pixel in the remote images.
5. Allow principals to share their workflows, applications (such as feature extraction and automatic segmentation) and algorithms to integrate the collection of disparate data sets from our diverse research domains.
6. Collect and catalog analysis results and research data from the investigators.
7. Allow investigators to search, access and view all data via the portal regardless of the locations of the repositories and resources.
8. Integrate related data and information from interdisciplinary studies into a single platform to facilitate collaboration, mash-ups, and highlight new findings.

A significant planetary geology community effort to combine different datasets from the same resolution occurred when the Mars Exploration Program committee put forward the MSL landing site workshops from 2009–2011. Many of the presentations advocating the final four sites (Holden crater, Mawrth Vallis, Eberswalde crater, and Gale crater) used a significant amount of manually co-registered images showing mineral volumes (Rice et al., 2013) in each site overlaying some topography. Since this effort is largely a manual process, different techniques have evolved using a combination of ENVI, ArcGIS, and other image editing software. The effort to orthorectify, geo-reference, co-register, and mosaic lunar images was performed using the NASA Integrated Software for Imagers and Spectrometers (ISIS) software (Rosiek et al., 2012). However, it would be much easier to map habitability and optimal places for exploration with co-registered Earth analog and Mars data in a community-accepted AIS system.

Ultimately, an AIS system capable of map and visualization functions will provide the context and the platform to locate, map, and predict where the “sweet spots” of habitable environments might occur (Figure 5, 6). A geospatial AIS model could describe the various disciplinary and integrated products (such as the ones produced by machine vision and probabilistic modeling), their metadata (types and definitions of information), and their attributes and relationships (such as geospatial resolution, sampling information, etc.).

## Summary

New exploration missions in search of extraterrestrial life should focus on authigenic minerals that form *in situ* to record water and environmental conditions of the sedimentary environment where life commonly flourishes. Astrobiological studies of terrestrial environments have revolutionized our assessment of the potential for life in surface to subsurface settings elsewhere in our solar system (e.g., Des Marais et al., 2008; Hays et al., 2017). Authigenic minerals of iron oxides, sulfates, clays, and carbonates are preserved on Mars as well as being common in terrestrial sedimentary systems. The Earth analogs can be used to constrain geochemical and environmental parameters of their formation (e.g., pH, temperature, pressure, aridity, etc.). and indicate the potential for life in watery environments.

Studies of any planetary habitable environments must examine the context and relationships across multiple scales of macro- (>km), to meso- (m), to microscale (<mm). Each nested scale requires: characterization of all physical, chemical and biological properties; and what environmental parameters might be important for assessing habitability. Quantifying biosignatures and habitability in Earth’s depositional and diagenetic environments requires understanding multiple scaled parameters that contribute to the geologic context.

The quantification of geospatial relationships and use of cybertechnology and computer vision mapping can be revolutionary for the astrobiology community. The computer vision can be a significant quantification of the visible correlates of habitability that will permit faster, more reliable, and more quantitative evaluation of geologic image samples, which is critical for integration of large data volumes. A new astrobiological information systems approach will allow astrobiology researchers to look at large and spatially diverse datasets to derive quantitative measures of where habitable environments exist.

It is clear that future exploration strategies to find extant life on other planetary bodies will always require maps with scaled regional to sub-mm targets that hone into the smallest scales of habitable environments that might possess biosignatures. Ultimately, a new standard of understanding habitability through geospatial maps can guide the search for the “sweet spots” where biosignatures exist and are best preserved. This has the strong potential to take planetary exploration for life to a new level. This can be a highly valuable time-saving advance that will allow faster and more intuitive science correlations and comparisons for both terrestrial and planetary datasets.

## Author Contributions

All authors listed have made a substantial, direct and intellectual contribution to the work, and approved it for publication.

## Conflict of Interest Statement

The authors declare that the research was conducted in the absence of any commercial or financial relationships that could be construed as a potential conflict of interest.

## Footnotes

1 https://trek.nasa.gov

2 https://trek.nasa.gov/moon/

3 https://trek.nasa.gov/mars/

4 https://trek/nasa.gov/vesta/

5 https://trek.nasa.gov/ceres/
