# Corrigendum: Exploring, Mapping, and Data Management Integration of Habitable Environments in Astrobiology

**DOI:** 10.3389/fmicb.2019.01190

**Published:** 2019-05-29

**Authors:** Marjorie A. Chan, Brenda B. Bowen, Frank A. Corsetti, William H. Farrand, Emily S. Law, Horton E. Newsom, Scott M. Perl, John R. Spear, David R. Thompson

**Affiliations:** ^1^Department of Geology and Geophysics, The University of Utah, Salt Lake City, UT, United States; ^2^Department of Earth Sciences, University of Southern California, Los Angeles, CA, United States; ^3^Space Science Institute, Boulder, CO, United States; ^4^Jet Propulsion Laboratory, California Institute of Technology, Pasadena, CA, United States; ^5^Department Earth and Planetary Sciences, University of New Mexico, Albuquerque, NM, United States; ^6^Department of Civil and Environmental Engineering, Colorado School of Mines, Golden, CO, United States

**Keywords:** astrobiology, habitable environments, authigenic minerals, Mars, cybertechnology, data management

“Scott M. Perl” was not included as an author in the published article and has now been added. All authors listed have made a substantial, direct and intellectual contribution to the work, and approved it for publication.

Additionally, due to the addition of the author Scott M. Perl, a correction has been made to the **Acknowledgments** section:

“A portion of this research took place at the Jet Propulsion Laboratory, California Institute of Technology, under a contract with the National Aeronautics and Space Administration. General ideas presented here developed out of various NASA and NSF grants to the authors, along with partial support from the NASA Astrobiology Institute (project: Rock Powered Life) to JS, NSF Coupled Natural Human Systems (Award #1617473) to BB, and NSF National Robotics Initiative (Award #IIS-1526667) for DT. We thank the reviewers for input that improved this manuscript.”

The authors apologize for this error and state that this does not change the scientific conclusions of the article in any way. The original article has been updated.

